# Government Recognition and Support for Family Caregivers: A Comparative Review of Policies in Northern and Southern Countries With a Focus on Oncology Caregivers

**DOI:** 10.7759/cureus.63085

**Published:** 2024-06-25

**Authors:** Hiba Bourissi, Soufiane Mellas

**Affiliations:** 1 Laboratory of Anatomy, Surgery, and Anesthesiology, Faculty of Medicine, Pharmacy, and Dental Medicine, Fez, MAR; 2 Health Sciences, Faculty of Science and Technology, Fez, MAR; 3 Urology, Centre Hospitalier Universitaire Hassan II, Fez, MAR

**Keywords:** cancer, southern countries, northern countries, role recognition, support policies, family caregivers

## Abstract

The recognition of informal caregivers by governments is gradually increasing. Currently, several countries, mainly in the Northern Hemisphere, have officially acknowledged the role of caregivers and offer various forms of support. In contrast, in southern countries, data on informal caregivers, especially those caring for cancer patients, remain sparse. Furthermore, the acknowledgment and support for caregivers’ roles are still lacking in many of these regions. Notably, existing policies in northern countries predominantly focus on caregivers for the elderly and disabled, with only a few initiatives directly supporting caregivers of cancer patients. This review aims to shed light on the measures some governments have taken to support caregivers and advocate for further actions and policies to enhance caregiver support. This review is grounded on a thorough examination of articles, governmental reports, and empirical studies related to family caregiver support.

## Introduction and background

Cancer continues to be a significant global public health challenge, affecting millions of individuals worldwide with profound social, psychological, and physical impacts [1,2]. In 2022 alone, it is estimated that there were approximately 19,965,054 new cases of cancer across all types, ages, and genders. Among these, lung and breast cancers were the most common, with incidence rates of 12.4% and 11.6%, respectively, followed by colorectal cancer at 9.6% [3]. Notably, cancer incidence is two to three times higher in developed countries than in developing ones [4]. Despite this, developing countries experience a significantly higher number of deaths from breast and cervical cancer, with women in these regions facing a 17% higher mortality rate than their counterparts in developed countries [4].

The impact of cancer extends beyond the patients to their family caregivers (FCGs), adversely affecting their health status and quality of life [1,2]. This issue is particularly acute in southern countries, where improving the quality of life for FCGs has become a top priority, like in Morocco. The National Plan for the Prevention and Control of Cancer in Morocco, for example, aims to enhance the quality of life for both patients and their FCGs by providing comprehensive support [5]. The third Sustainable Development Goal focuses on health and well-being, aiming to “Ensure healthy lives and promote well-being for all at all ages” [6].

The crucial role of the family in oncology care ensuring continuity of care and social support is well documented, highlighting the significant socioeconomic, professional, and psychological impact of cancer on FCGs, sometimes even more so than on the patients themselves [7]. In this context, a comprehensive literature review was conducted to describe the national policies for supporting FCGs, focusing on comparisons between countries in the northern and southern hemispheres. Specific objectives of this review include identifying the policies recognizing FCGs and formulating recommendations to improve support for FCGs, specifically those of patients with cancer.

## Review

Methodology

*The Objective of the Review*

The objective of the review is to synthesize the support policies for FCGs from a comparative perspective between northern and southern countries. It aims to provide a narrative analysis based on a selection of articles, government reports, and available studies, highlighting existing policies and documented initiatives and identifying gaps in policies.

Nature of the Review and Methodological Approach

This review adopts a descriptive approach to offer a general overview of the support policies for FCGs, relying on the authors’ expertise and judgment. It prioritizes the establishment and ongoing relevance of policies over the recency of data.

While the review does not follow a strict protocol or standardized methodology, it is inspired by a methodical approach to the selection of sources. The sources were chosen based on their relevance to illustrate support policies for FCGs in various geographical and socioeconomic contexts.

Two strata were established for northern and southern countries to include articles discussing support policies as well as gaps in policies. The goal is to highlight both initiatives and gaps in support for FCGs. The review involved searching academic databases and governmental sites using keywords related to FCGs and support policies in both northern and southern countries.

The review recognizes the variation in timelines for establishing and updating policies among different countries. It focuses on the content and effectiveness of policies rather than their specific periods of implementation. Articles included in the review are from periods when policies were first established, especially in countries with long-standing policies without significant updates since then. *Inclusion and Exclusion Criteria*

Inclusion criteria encompassed articles in English, French, and Spanish discussing support policies, government reports, and documents providing insights into support policies for FCGs in northern and southern countries. Exclusion criteria involved documentation not addressing FCGs, redundant articles, and non-relevant documents.

The review involved searching academic databases (PubMed, Scopus, Google Scholar) and governmental sites using keywords related to FCGs and support policies in both northern and southern countries.

Review Analysis

The review focuses on describing and comparing policies without a detailed statistical analysis. It aims to offer an overview of support strategies for FCGs and highlight regional differences based on available publications, presenting results narratively for clarity and coherence.

The situation in northern countries

Support for FCGs varies from one country to another. The European Union has committed to supporting FCGs by implementing a set of measures. Among these are allowances, tax exemptions, legal coverage for working caregivers, and integration of social and health services to assist FCGs in hospitals, among others [8]. The role of the FCG is officially recognized by several countries of the European Union, including Austria, Belgium, Germany, Denmark, Spain, Finland, France, Hungary, Ireland, Italy, Luxembourg, the Netherlands, Sweden, and the United Kingdom, which was previously part of the European Union [9].

France

In France, the concept of FCG emerged in 2005 with the law of February 11th recognizing the caregiver of a disabled person, and the recognition of the caregiver’s role was established in the law of August 8th, 2016 [10].

Currently, there are caregiving leave options for individuals assisting a relative with a loss of autonomy since the law of August 21st, 2003. These include the family solidarity leave and the caregiver leave, both of which are unpaid by the employer (L. 3142-16 of the Labor Code) [11]. The former aims to assist a relative suffering from a disease with a poor prognosis or an incurable or serious illness in an advanced or terminal stage, and the latter aims to accompany a disabled relative or one with a significant loss of autonomy (disability rate of at least 80%) [12]. A daily caregiver allowance has been in place since September 30th, 2020, set at 43.83 euros and 52.08 euros per day for individuals in a partnership or living alone, respectively, with a ceiling of 66 euros per day [10]. There is also a daily allowance for supporting a person at the end of life, set at 56.33 euros per day (L. 168-1 et seq., D. 168-1 et seq., circular DSS of March 24th, 2011) [11,12]. From January 1, 2024, the caregiver allowance is set at 64.54 euros per full day and 32.27 euros for a half-day. This allowance can be claimed for a maximum of 22 days per month and must not exceed 66 days over the caregiver’s professional career. The allowance days can be taken as half-days, within the monthly limit of 22 days [13].

However, not all FCGs of cancer patients are directly eligible for the daily support measures from the Personal Assistance (PA) system, as it is only offered to caregivers of individuals with disabilities, those experiencing significant loss of autonomy (with a disability rate of at least 80%) [10], or those in end-of-life care [12]. The establishment of a specific allowance for all FCGs of cancer patients remains a significant expectation of the French League Against Cancer [14].

Regarding the respite for caregivers, there are support organizations and nursing care services that mobilize at home, performing various household tasks such as meal preparation, assistance with going to bed or waking up, personal hygiene, changing clothes, and medication [12]. There is also a relief option that allows the caregiver to be replaced by a health professional for several consecutive days [12]. Recently, a system has been implemented where employees can donate their rest days to a colleague who is a parent of a severely ill child or a caregiver, allowing them to be paid during this leave [11].

Belgium

In Belgium, the legal recognition of the caregiver’s status was established [15]. Now, it has become possible to have caregiver status and benefit from several social rights, notably the paid leave called “thematic leave” starting from September 1, 2020 [15,16]. At the local level, associations and online platforms play a very important role in offering support actions, awareness-raising, respite offers, etc. [15]. The caregiver can have access to respite care aimed at supporting and accompanying the caregiver and to home help services in their region. They also benefit from counseling and psycho-educational support [15].

United Kingdom

The United Kingdom has implemented a public strategy to support caregivers, the National Strategy for Carers, with several financial support programs, such as the “carer’s allowance,” which compensates for lost income, and “income support,” which provides an additional allowance [17]. Caregivers are also entitled to unpaid leave of a duration defined by their employer and flexibility in working hours [17].

Spain

In Spain, the “*Prestación económica para cuidados en el medio familiar y apoyo a cuidadores no profesionales*” aims to supporting FCGs of highly dependent persons. Caregivers can receive between 390 and 487 euros and must participate in training programs [17].

Canada

In Canada, there is an employment insurance program that allows employees to take time off to care for a sick relative, with support of up to 55% of their salary up to a maximum of 668 dollars per week [18]. The program distinguishes between caregivers for children, adults, and compassionate caregivers, offering a maximum number of paid weeks up to 35 weeks for child caregivers, 15 weeks for adult caregivers, and 26 weeks for compassionate caregivers [18]. Healthcare benefits provided by a health professional (doctor and/or nurse) have been made available to caregivers for 52 weeks following the declaration of the relative’s illness [18].

United States

In the United States, a law was enacted in 2018 called the *RAISE FCGs Act* aimed at recognizing, involving, and supporting FCGs [19]. It calls on the Department of Health and Human Services to develop a support strategy amounting to 43 million dollars for FCGs [17]. American FCGs are also entitled to unpaid leave not exceeding 12 weeks [17].

The situation in southern countries

Saudi Arabia

The Saudi caregiver, like their counterparts in other countries, faces several difficulties, including social and financial challenges, and requires a stable allowance. Caregivers find themselves facing these challenges alone or receiving support only from their immediate circle [20]. Despite their daily commitment to supporting their sick relative, the role of the caregiver remains unrecognized by the Saudi state [20].

Iran

In Iran, the role of the caregiver is not recognized either. Despite the significant role caregivers play in keeping cancer patients at home, they are not fully and adequately integrated into the Iranian healthcare system or care programs [21]. This lack of support for Iranian caregivers and addressing their needs has been reported in several studies [22]. In this regard, it has been suggested to integrate caregivers into Iranian cancer care programs and develop a comprehensive program focused on the families of cancer patients based on their overall needs [21].

Brazil

In Brazil, there are no specific initiatives dedicated to caregivers, with a total absence of public policies for the protection and support of Brazilian caregivers [8].

Turkey

There is no support program specifically for caregivers of cancer patients in Turkey. However, according to The Aging Readiness & Competitiveness Initiative, the government established in 2006 a support program for caregivers of the elderly titled the “Caregiver Service Program,” which aims to subsidize low-income caregivers who dedicate at least eight hours to caring for their elderly relative and receive a monthly salary equivalent to 280 USD [23].

Singapore

In the same way, in Singapore, there are no support strategies specifically aimed at caregivers, and there is an urgent need to develop a comprehensive support system that encompasses both the patient with advanced-stage cancer and their caregiver [24].

Algeria and Tunisia

Likewise, in Algeria and Tunisia, there are no support policies for caregivers of cancer patients. However, there is assistance available for the descendants of an elderly person who is losing autonomy and lacking financial resources, as evidenced by Algeria’s law No. 10-12 of 23 Muharram 1432 corresponding to December 29, 2010, and Tunisia [25].

Morocco

**Figure 1:**
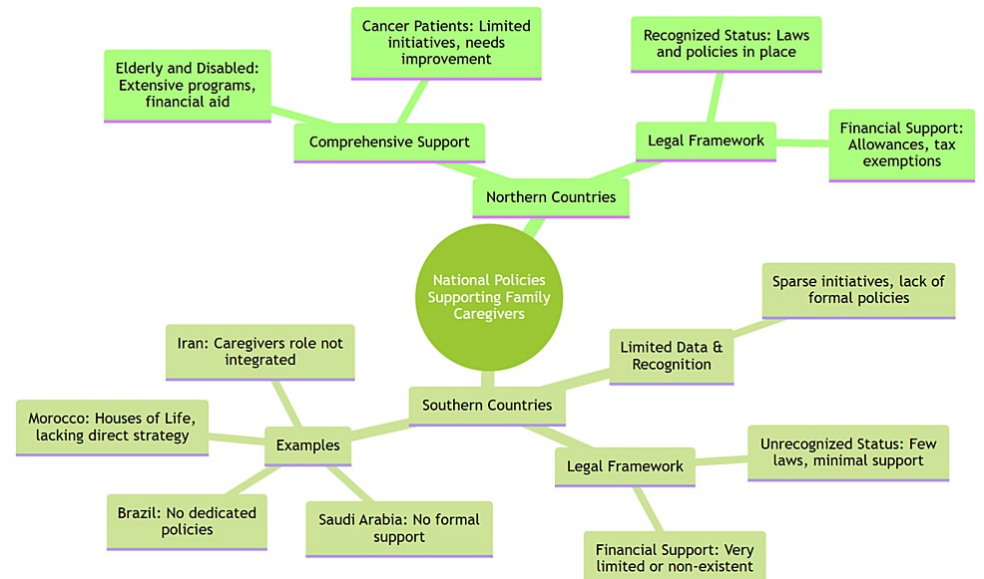
Summary diagram of the key elements of support strategies for family caregivers in northern versus southern countries.

In Morocco, oncology patients always arrive at care units accompanied by at least one family member, who comes to assist and support them, proving to be very beneficial for the patient [26]. Supporting the cancer patient and their caregiver is one of the main objectives of the Lalla Salma Foundation for Cancer Prevention and Treatment. The foundation has made available “Maisons de Vie” (Houses of Life), which are temporary housing facilities located near oncology centers to accommodate the patient and their caregiver [5]. To date, there are two Houses of Life in Casablanca for children and adults, with others located in Rabat, Fez, Marrakech, Meknes, Agadir, and Tangier [5]. These Houses of Life have a multidisciplinary staff, including psychologists, health professionals (doctors/nurses), social workers, and volunteers [5]. Complementary sociocultural activities are organized, such as musical evenings, dinners, and outings [5]. The role of the FCGs in Morocco is not recognized, and the country lacks a direct and specific strategy to support them, particularly those caring for cancer patients. This review shows that northern countries have set up more organized support systems for FCGs, including monetary help, legal protection, and respite services. In contrast, in southern countries like Brazil, Turkey, and Iran, there is a clear lack of actions and policies for FCGs (Figure 1). This may be because family care has always been a part of the cultural expectations in these countries. This difference could highlight how social and cultural norms affect the development and execution of support policies for caregivers.

Discussion

Countries in the north have strong health policies with significant budgets, leading to highly effective health systems. Conversely, southern countries often experience less effective health systems [27,28]. This difference extends to FCG support policies, where northern countries offer more structured systems providing financial assistance, legal protection, and respite services. On the other hand, countries like Brazil, Turkey, and Iran in the south lack well-defined policies for FCGs, often influenced by cultural norms that prioritize family care over institutional support. This variation in caregiver support highlights how cultural norms impact the effectiveness of health policies.

Effective health policies must consider cultural contexts, especially in southern nations where culture plays a crucial role in shaping health beliefs and practices. Incorporating these contexts can help tailor health policies to suit the specific needs of populations better [29]. Discrepancies in caregiver support policies between northern and southern countries may stem from cultural norms that favor family-based care in the south, leading to a reluctance to institutionalize caregiver support, as seen in the north. Table 1 illustrates these differences in policy recognition, types of support, and governmental involvement, highlighting both the initiatives and gaps in support for FCGs across these regions.

**Table 1:** Comparative overview of family caregiver support policies in northern and southern countries.

Aspect	Northern countries	Southern countries
Policy recognition	Many countries officially recognize the role of family caregivers	Limited official recognition of family caregivers
Types of support	Allowances and financial support (e.g., France, United Kingdom). Legal protections (e.g., Belgium, United Kingdom). Respite services and professional support (e.g., France, Belgium). Flexible working hours and unpaid leave (e.g., United Kingdom, Canada)	Very limited or no financial support (e.g., Brazil, Iran). Social and financial challenges are largely unsupported (e.g., Saudi Arabia). Limited professional support and respite services. Cultural expectations often place caregiving responsibilities solely on families
Specific policies for cancer caregivers	Few specific policies; generally integrated with policies for caregivers of the elderly and disabled (e.g., France)	Generally no specific policies for cancer caregivers (e.g., Turkey, Iran)
Support organizations	Numerous support organizations and programs are available (e.g., local associations in Belgium, and national strategies in the United Kingdom)	Very few support organizations; reliance on family and informal networks (e.g., Saudi Arabia).
Governmental involvement	High level of governmental involvement and structured support programs (e.g., National Strategy for Carers in the United Kingdom).	Low level of governmental involvement; limited public policies specifically addressing caregiver needs (e.g., Brazil, Turkey).
Examples of initiatives	France: Caregiving leave, daily allowances, respite services. United Kingdom: Carer’s allowance, income support, unpaid leave, flexible working hours. Belgium: Paid thematic leave and local support associations	Morocco: Houses of Life near oncology centers by the Lalla Salma Foundation. Saudi Arabia: Informal support within immediate circles. Turkey: Caregiver Service Program for the elderly, but none for cancer caregivers. Iran: Lack of integration into healthcare and support programs

The COVID-19 pandemic emphasized the importance of adapting culturally sensitive policies to address emerging health crises. Throughout the pandemic, the heightened responsibilities of FCGs for individuals with chronic illnesses or disabilities underscored the need to enhance healthcare systems to better assist them, ensuring access to care during health emergencies [30]. The pandemic revealed immediate challenges and shed light on underlying shortcomings in caregiver support policies.

Nevertheless, many caregiver-focused policies tend to prioritize patient care over meeting the comprehensive needs of caregivers themselves. This emphasis exposes flaws in support policies and emphasizes the necessity for a thorough reevaluation of existing measures [31]. Recognizing these deficiencies is the initial step toward reforming support systems, requiring a broad and innovative approach to policy development. Existing policies in northern countries predominantly target caregivers for the elderly and disabled, with limited programs directly addressing the needs of oncology caregivers, indicating an area for potential policy improvement globally.

Developing effective support policies for caregivers necessitates acknowledging their diverse needs, integrating informal care into the formal healthcare system, and adopting a forward-thinking, inclusive approach. These policies should aim to enhance the well-being of individuals and communities, as well as manage resources efficiently both now and in the future [32]. Implementing non-financial interventions such as education and respite care, which have proven beneficial in European contexts, is part of this strategy [33]. These interventions underscore the importance of a holistic approach to support policy, considering the intricate interplay of social, cultural, and economic factors influencing caregiver experiences worldwide.

In summary, when comparing FCG support policies between northern and southern countries, noticeable distinctions become apparent. Northern nations typically have well-established policies that officially acknowledge caregivers, offering financial aid, legal safeguards, and professional assistance. Conversely, southern countries often exhibit limited official acknowledgment and assistance for caregivers, with minimal government intervention and reliance on family-centered care influenced by cultural traditions. Nonetheless, both regions are gradually recognizing the significance of caregiver support, though the implementation varies, focusing on different aspects, especially concerning the specific needs of caregivers for cancer patients.

The analysis underscores the urgent need for southern countries to establish more comprehensive support systems for all FCGs, particularly those caring for cancer patients, to improve the quality of life for both patients and caregivers.

Strengths and limitations

This review provides an extensive overview of support policies for FCGs across various geographical regions, incorporating a wide range of sources to offer a comprehensive perspective on the subject. The methodology used for selecting sources aims to minimize bias and ensure a balanced representation of both northern and southern countries.

However, there are some limitations to this approach. The descriptive nature of the review may introduce subjectivity into the analysis. By limiting the inclusion of articles to English, French, and Spanish languages, potentially relevant studies in other languages could be left out. Furthermore, the availability of data from certain regions may be restricted, impacting the comparability of support policies across different areas.

## Conclusions

Becoming an FCG is often challenging, and this new role generates needs that, if unmet, can have impacts on multiple levels. With improvements in oncology care and the trend toward home care, the role of the caregiver can extend for several months or even years, causing a change in daily life activities and requiring a full-time commitment. To date, despite the increasing demand, there are few resources specifically established to support caregivers of cancer patients. Health professionals, doctors, and nurses, in turn, can contribute to improving the situation of caregivers by meeting their needs, especially in terms of information, communication, and home care practices.

By addressing these challenges, southern countries, including Morocco, must invest in supporting caregivers, who are an important resource, and meet their needs by adopting new health policies that encompass the caregiver. Policymakers can learn from the experience of northern countries to take this step, recognize the role of the caregiver, and provide support. Such initiatives are vital not only for enhancing caregiver well-being but also for strengthening the overall efficiency and effectiveness of healthcare delivery
